# Inflammation and oxidative stress in heart failure: effects of exercise intensity and duration

**DOI:** 10.1590/1414-431X20176393

**Published:** 2017-08-07

**Authors:** G.A. Ribeiro-Samora, L.A. Rabelo, A.C.C. Ferreira, M. Favero, G.S. Guedes, L.S.M. Pereira, V.F. Parreira, R.R. Britto

**Affiliations:** 1 Programa de Pós-Graduação em Ciências da Reabilitação, Universidade Federal de Minas Gerais, Belo Horizonte, MG, Brasil; 2 Laboratório de Reatividade Cardiovascular, Instituto de Ciências Biológicas e da Saúde, Universidade Federal de Alagoas, Maceió, AL, Brasil; 3 Departamento de Fisioterapia, Universidade Federal de Minas Gerais, Belo Horizonte, MG, Brasil; 4 Faculdade de Nutrição, Universidade Federal de Alagoas, Maceió, AL, Brasil

**Keywords:** Heart failure, Inflammation, Oxidative stress, Exercise intensity, Exercise duration

## Abstract

Although acute exercise is apparently pro-inflammatory and increases oxidative stress, it can promote the necessary stress stimulus to train chronic adaptations in patients with chronic heart failure (CHF). This study aimed to compare the effects of exercise intensity and duration on the inflammatory markers soluble tumor necrosis factor receptor (sTNFR1) and interleukin-6 (IL-6), and on oxidative stress [malondialdehyde (MDA) and antioxidant enzymes: catalase (CAT) and superoxide dismutase (SOD)] in individuals with CHF. Eighteen patients performed three exercise sessions: 30 min of moderate-intensity (M30) exercise, 30 min of low-intensity (L30) exercise, and 45 min of low-intensity (L45) exercise. Blood analysis was performed before exercise (baseline), immediately after each session (after), and 1 h after the end of each session (1h after). Thirty min of M30 exercise promoted a larger stressor stimulus, both pro-inflammatory and pro-oxidative, than that promoted by exercises L30 and L45. This was evidenced by increased sTNFR1 and MDA levels after exercise M30. In response to this stressor stimulus, 1 h after exercise, there was an increase in IL-6 and CAT levels, and a return of sTNFR1 to baseline levels. These findings suggest that compared with the duration of exercise, the exercise intensity was an important factor of physiologic adjustments.

## Introduction

Heart failure is a progressive syndrome, characterized by exercise intolerance, dyspnea, fatigue, and decrease in quality of life resulting from the inability of the heart to maintain a cardiac output sufficient to meet tissue demands (1,2). It is a relatively common clinical condition, and is the final stage of several forms of cardiovascular disease (3).

The deteriorated heart function, *per se,* is able to modulate the inflammatory response and the production of reactive oxygen species (ROS). In heart failure, tissue hypoxia caused either by low cardiac output or by sympathetic vasoconstriction may also trigger an increase in the production of free radicals (4), which is a potent stimulus for the synthesis of pro-inflammatory cytokines such as tumor necrosis factor-alpha (TNF-α) and interleukin-6 (IL-6) (5–7). Inflammation can also induce oxidative stress. Thus, oxidative stress and inflammation are involved in a self-perpetuating cycle (8,9).

Acute exercise is apparently pro-inflammatory and increases oxidative stress; however, it can produce the necessary stress to stimulate chronic adaptations that are beneficial (10), including the reduction of inflammatory response (1,11) and oxidative stress by enhancing the antioxidant system (12). These exercise-mediated responses seem to be dependent on several factors, such as exercise intensity, duration, and the muscle mass involved (13). These responses have barely been studied in patients with chronic heart failure (CHF). In a previous study on patients with CHF, we observed that 30 min of moderate-intensity exercise elicited a better positive acute inflammatory response than that elicited by mild exercise (14). However, it is not clear whether this result is related to the intensity, duration, or the amount of exercise. Thus, this study aimed to compare the effects of three exercise situations of different intensities and durations on the levels of the inflammatory markers sTNFR1 (soluble tumor necrosis factor receptor) and IL-6, and on oxidative stress, as assessed by plasma malondialdehyde (MDA) concentrations, as well as the activity of the antioxidant enzymes catalase (CAT) and superoxide dismutase (SOD), in individuals with CHF.

## Material and Methods

This study was approved by the Ethics Committee of the Universidade Federal de Minas Gerais (protocol No. 050/09) in which the research was conducted, in accordance with the principles outlined in the Declaration of Helsinki. All volunteers provided a written informed consent for participation.

### Subjects

Eighteen individuals (aged 28–59 years) from the Heart Failure Center of the University Hospital participated in the study. All participants were clinically diagnosed with heart failure of New York Heart Association (NYHA) functional class I to III. Patients were included if they had predominantly systolic heart failure (left ventricular ejection fraction <45%), were clinically stable for at least 2 months prior to the start of the study, and were undergoing optimized pharmacological treatment. The exclusion criteria were obesity (body mass index ≥30 kg/m^2^); use of anti-inflammatory and immunosuppressive agents or vitamins A, C, or E; presence of other inflammatory disease, kidney failure, or orthopedic or neurologic dysfunctions limiting physical exercise; history of pulmonary disease, unstable angina, uncontrolled arrhythmias, or peripheral arterial disease; regular physical exercise; and inability to reach a respiratory exchange ratio ≥1.1 during maximal cardiopulmonary exercise testing (CPET).

### Procedures

On the first experimental day, the volunteers visited the laboratory for an initial assessment that included medical history, anthropometric measures, and CPET, as described below.

### Cardiopulmonary exercise testing

Maximal CPET was performed on an electronic treadmill (Millenium Classic CI®; Inbramed/Inbrasport, Brazil), with analysis of expired gases using the breath-by-breath method with a metabolic cart (Medical Graphics® CPX Ultima; USA) and a ramping protocol that increased the velocity and/or slope every 10 s, until fatigue. None of the tests needed to be interrupted by the researchers. The peak oxygen uptake (VO_2_peak) was determined using the average of the oxygen uptake in the last 30 s (15).

Arterial blood pressure was measured using the auscultation method, subjective effort perception was estimated using the modified Borg scale (16), peripheral oxygen saturation was measured using a pulse oximeter (Mediaid Model 300 Series; Mediaid Inc., USA), and the heart rate was continuously recorded using an electrocardiogram (Cardioperfect; Welch Allyn, Inc., USA).

For CPET, the temperature was maintained between 18° and 22°C, and the relative humidity varied between 40 and 60%. Barometric pressure was measured to ensure gas measurement under standardized conditions. The subjects were instructed to continue their usual medication, to refrain from cigarettes and caffeinated food or drinks for 3 h prior to the test, and to avoid physical exercise for 12 h prior to the test (17,18).

### Exercise situations

The subjects returned to the laboratory between 7:00 and 10:00 am after an overnight fast of 9–12 h, no longer than 2 weeks from the initial assessment. They were instructed not to perform any physical exercise for 24 h before the 3 days of submaximal testing and to complete a detailed food diary during the study period.

After a 10-min rest, the first (pre-exercise) blood sample was collected. The subjects then received a standard breakfast of bread, butter, mozzarella cheese, commercial grape juice, coffee, and banana. This breakfast was *ad libitum* but was documented in order to guarantee an equivalent type and volume of food intake on the other test days.

In random order and separated by a 3- to 7-day interval, the subjects performed three bouts of submaximal, continuous aerobic exercise on an electronic treadmill: a moderate-intensity walk, with 60% of the peak heart rate achieved at the CPET in 30 min (exercise M30); a low-intensity walk, with 40% of the peak heart rate achieved at the CPET heart rate corresponding to 40% of VO_2_peak in 30 min (exercise L30); and a low-intensity walk, with 40% of the peak heart rate achieved at the CPET in 45 min (exercise L45, isocaloric to exercise M30) (19). For all three situations, the volunteers performed active warm-up and cool-down phases of three min each, at an intensity corresponding to the initial velocity and slope of the treadmill during CPET, in addition to the time specified for each exercise.

For all exercise situations (L30, L45, and M30), in addition to the pre-exercise blood sample (baseline), two other samples were collected: at the end of the exercise (after) and 1 h after the end of the session (1 h after).

### Food diary

To avoid any influence of food on physiologic markers, the subjects were instructed to maintain their usual eating habits during the study and to complete a food diary with a description of the type, amount, and time of food intake for three days before the first submaximal exercise. They received a copy of the first day’s food report and were instructed to repeat the same diet on the other 2 days.

### Blood collection and analysis

At each time (baseline, after, and 1 h after) and for each experimental situation (L30, M30, and L45), approximately 10 mL of blood was drawn from the antecubital vein, out of which 4 mL was collected in tubes containing ethylenediamine tetraacetic acid (EDTA), and 4.5 mL in tubes containing citrate. The tubes were centrifuged (Fanem®, Brazil) immediately after each blood collection, in order to separate plasma and erythrocytes. Those with citrate were centrifuged at 1,500 *g* for 15 min at room temperature and those with EDTA were spun at 1,300 *g* in a refrigerated centrifuge (B. Braun Sigma-Aldrich 2K15, Germany) at 4°C for 10 min. Immediately after centrifugation, plasma and erythrocytes were separated into 1.5-mL sterile microtubes (Axygen®, USA) in a laminar flow hood and placed in a freezer (ColdLab CL374-80; Brazil) at -80°C.

### Inflammation

Since TNF-α has a short half-life, the soluble receptor sTNFR1 was chosen to indicate the activity of TNF-α, because it provides more stable serum levels (20).

Concentrations of IL-6 and sTNFR1 were measured using an enzyme-linked immunosorbent assay (ELISA), according to the manufacturer's specifications (Quantikine High Sensitivity Human for IL-6 and Quantikine Human sTNF RI/TNFRSF1A Immunoassay for sTNFR1; R&D Systems, USA).

### Oxidative stress

The concentrations of MDA, CAT, and SOD were assessed using the methods described below. The formation of MDA, an indicator of lipid peroxidation, was assessed by the detection of thiobarbituric acid-reactive substances, according to the method described by Wallin et al. (21). The CAT activity was determined by evaluating the decrease in the index of a reaction containing a phosphate and hydrogen peroxide tampon (22). The SOD enzyme activity was measured using a commercial kit (Fluka; Sigma-Aldrich, Germany).

### Statistical analysis

The normality of the continuous variables was assessed by applying the Shapiro-Wilk test. Nonparametric statistics were used because the variables were not normally distributed. The changes (delta) from after exercise to baseline (delta after) and from 1 h after exercise to after exercise (delta 1 h after) were calculated and the differences between the 3 submaximal exercises (L30, M30, and L45) were assessed using Friedman's test for delta values (in 3×2 comparisons), followed by the Wilcoxon *post hoc* test. The differences in the plasma levels (baseline, after, and 1 h after) of intra-submaximal exercises were assessed using Friedman's and Wilcoxon's tests. A P value <0.05 was considered to be statistically significant. The physical and clinical characteristics are reported as the proportion of patients or the mean and standard deviation (SD). Other variables are reported as median values and interquartile range. All data were analyzed using the Statistical Package for the Social Sciences for Windows version 19.0 (SPSS, Inc., USA).

## Results

Table 1 shows the physical and clinical characteristics of the study participants (n=18).

**Table 1.** Physical and clinical characteristics of individuals with heart failure.

Characteristics	Values
Age (years)	45.44±11.26
Men/women (n)	13/5
Body mass index (kg/m^2^)	25.32±4.04
VO_2_peak (mL·kg^-1^·min^-1^)	25.61±6.11
LVEF (%)	36.67±10.28
Etiology (%)	
Idiopathic	28
Ischemic	22
Hypertensive	22
Valvulopathy	17
Others	11
Medications, n (%)	
Beta blocker	18 (100)
Diuretic	13 (72)
Digitalis	12 (66)
ACEi/ARB	15 (83)
Amiodarone	14 (77)
NYHA class, n (%)	
NYHA I	7 (39)
NYHA II	7 (39)
NYHA III	4 (22)

Data are reported as means±SD, for n=18. VO_2_peak: oxygen output achieved in the peak exercise; LVEF: left ventricular ejection fraction; ACEi/ARB: angiotensin-converting enzyme inhibitor/angiotensin-receptor blocker; NYHA: New York Heart Association.

### Inflammation

Table 2 shows the results for intra-submaximal exercise analysis (absolute data). The IL-6 levels increased after L30 (P=0.014) and returned to baseline levels 1 h after exercise. In M30 and L45, no significant differences were observed (P<0.05). The sTNFR1 levels increased after M30 exercise (P=0.039) and returned to baseline levels 1 h after exercise.

**Table 2.** Absolute values and comparisons of IL-6, sTNFR1, MDA, CAT and SOD levels between intra-exercise of low intensity for 30 min (L30), moderate intensity for 30 min (M30) and low intensity for 45 min (L45).

Outcomes	L30			M30			L45		
	Baseline	After	1 h after	Baseline	After	1 h after	Baseline	After	1 h after
IL-6 (pg/mL)	1.53 (0.58)	**2.19** * (2.05)	**1.50** † (0.40)	1.33 (1.10)	1.17 (0.94)	1.32 (2.01)	1.36 (1.20)	1.18 (0.85)	1.25 (0.89)
sTNFR1 (pg/mL)	694.99 (512.76)	675.32 (614.69)	676.64 (650.75)	787.69 (633.30)	**842.88** * (558.75)	**717.44** † (636.44)	716.65 (609.73)	743.27 (554.79)	756.78 (524.65)
MDA (nmol/mg protein)	3.03 (2.07)	2.93 (1.62)	3.13 (1.62)	2.07 (1.54)	**2.30** * (1.92)	**3.33** * † (3.51)	2.30 (1.78)	**3.16** * (4.72)	2.90 (2.05)
CAT (µ·min^-1^˙mL^-1^/mM Hb)	12.37 (1.23)	12.88 (2.21)	12.76 (2.47)	12.93 (4.92)	12.71 (4.12)	**13.86** * (3.77)	13.37 (4.11)	14.09 (5.22)	12.73 (4.02)
SOD (IU/mM Hb)	1.24 (0.42)	1.21 (0.37)	1.41 (0.33)	1.04 (0.74)	1.04 (0.69)	1.07 (0.58)	1.11 (0.36)	1.19 (0.65)	1.07 (0.78)

Data are reported as median (interquartile range).

* P<0.05 compared with baseline;

† P<0.05 compared with after (Friedman test followed Wilcoxon *post hoc* test). Data reported in bold are significantly different. IL-6: interleukin-6; sTNFR1: soluble tumor necrosis factor receptor; MDA: malondialdehyde; CAT: catalase; SOD: superoxide dismutase.

**Figure 1.**
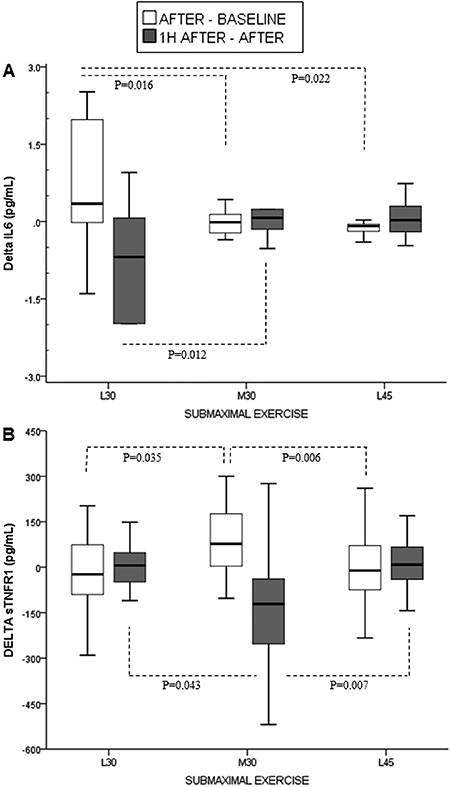
Delta values of inflammatory markers at AFTER (immediately after) to BASELINE and 1H AFTER to AFTER assessed for moderate exercises of intensity for 30 min (M30), low intensity for 30 min (L30) and low intensity for 45 min (L45). *A*, interleukin-6 (IL6, pg/mL); *B*, soluble tumor necrosis factor receptor (sTNFR1, pg/mL). Data are reported as median values and interquartile range. Statistical analysis was done with Friedman's test followed by the Wilcoxon *post hoc* test.

Figure 1 (panels A and B) shows the delta comparisons of the effects of exercises M30, L30, and L45 on inflammatory markers IL-6 and sTNFR1. The delta IL-6 increased after exercise L30 *vs* M30 (P=0.016) and L30 *vs* L45 (P=0.022). However, samples from 1 h after exercise showed a decrease in delta IL-6 for L30 compared with M30 (P=0.012). There was no significant difference between M30 and L45 (P<0.05). The delta sTNFR1 after M30 increased in relation to L30 and L45 (P=0.035 and P=0.006, respectively), and 1 h after M30 these deltas reduced in relation to L30 (P=0.043) and L45 (P=0.007). There was no significant difference between the deltas for L30 and L45 (P<0.05).

### Oxidative stress

Table 2 shows absolute values and the results of comparisons between intra-exercise MDA, CAT, and SOD levels. The MDA levels with M30 increased after (P=0.001) and 1 h after (P=0.004) and with L45, increased after (P=0.010). CAT levels significantly increased 1 h after M30 (P=0.049).

**Figure 2.**
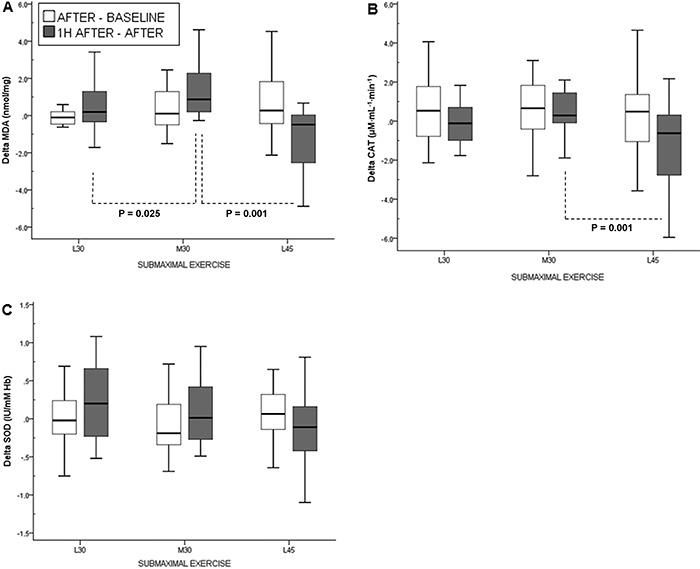
Delta values of oxidative stress and antioxidant markers at AFTER (immediately after) to BASELINE and 1 H AFTER to AFTER assessed for exercise of moderate intensity for 30 min (M30), low intensity for 30 min (L30) and low intensity for 45 min (L45). *A*, malondialdehyde (MDA, nmol/mg protein); *B*, catalase enzyme (CAT, µM·min^–1^·mL^–1^/mM Hb); *C*, superoxide dismutase (SOD, IU/mM Hb). Data are reported as median values and interquartile range. Statistical analysis was done with Friedman's test followed by the Wilcoxon *post hoc* test.

The delta MDA levels 1 h after M30 were higher than those after L45 (P=0.001) and L30 (P=0.025), and also higher 1 h after L45 *vs* L30 (P=0.008; Figure 2A). In addition, delta CAT values 1 h after exercise M30 increased in relation to L45 (P=0.001) but were not different from those 1 h after L30 (P<0.05; Figure 2B). There was no significant change in delta SOD between exercises L30, M30, and L45 (Figure 2C).

## Discussion

The main result of this study was that 30 min of moderate-intensity exercise promoted a higher stressor stimulus, both pro-inflammatory and pro-oxidative, than that promoted by low-intensity exercises during 30 or 45 min, suggesting that intensity was more responsible for the response than duration. This was evidenced by increased sTNFR1 and MDA levels after M30 exercise. In response to this stressor stimulus, there was an increase in CAT levels and a return of sTNFR1 to baseline levels 1 h after exercise, which demonstrates the anti-inflammatory action of IL-6 inhibiting the expression of TNF-α as well as the activation of the enzyme antioxidant system, as expressed by the increase in CAT.

Studies in different populations (23–26) have reported an anti-inflammatory response immediately after a single exercise session, with an increase in plasma IL-6 and sTNFR1 levels, including in CHF patients. In a previous study, (14) we compared the acute response of IL-6 and sTNFR1 to that of two submaximal intensity exercises, low and moderate, in individuals with heart failure (NYHA class II and III), and observed an increase of sTNFR1 after 30 min of moderate-intensity exercise, which returned to the baseline 1 h after the activity. However, levels of IL-6 significantly increased at 1 h after 30 min of exercise at a moderate intensity only. These results are inconsistent with the results of the present study; one of the reasons for the inconsistency could be the different NYHA classes that constituted the samples. In this study, 78% of the patients belonged to NYHA classes I and II, whereas in the study by Pereira et al. (14) about half of the sample comprised patients with NYHA class III heart failure. Further, baseline levels of IL-6 were higher in Pereira et al. (14) study than those observed in this study. It is known that serum levels of IL-6 are directly associated with worsening of NYHA functional class, especially when associated with reduced left ventricular ejection fraction and peak oxygen consumption (27).

The mechanisms that explain the increase in exercise-induced pro-inflammatory cytokine levels in patients with CHF are not well established in the literature, and some hypotheses have included adrenergic stimulation and peripheral tissue hypoperfusion (5,6). TNF-α and IL-6 are early inflammation mediators and act in an interconnected manner, since TNF-α induces the production of IL-6, which subsequently inhibits TNF-α expression (26,28). Although with the results of the present study we cannot establish a cause-and-effect relationship between oxidative stress and inflammation, we hypothesized that the observed increase in MDA and sTNFR1 levels immediately after M30 exercise could be mediated by the production of the superoxide anion and hydrogen peroxide free radicals, stimulating the release of TNF-α and promoting lipid peroxidation (9). Some studies (29,30) have reported that physical exercise can promote an immediate inflammatory response marked by leukocytosis, increased production of reactive oxygen species, and increased C-reactive protein levels. The benefits of this stress would be adaptations such as increased antioxidant enzymatic activity and decreased chronic inflammation (31–33).

The physiologic adjustments in response to inflammation and oxidative stress are integrated (9). Physiological levels of ROS are pivotal for force production in skeletal muscle. In addition, high levels of exercise-induced ROS, produced through different routes, such as calcium-dependent signaling and mitogen activated protein kinase (MAP kinase), as well as several tyrosine kinase cascades and antioxidants defenses failure, induce muscle dysfunction, probably by fatigue and/or atrophy (33,34). Oxidative stress stimulates the production of cytokines and adhesion molecules, and the activation and proliferation of lymphocytes through activation of the kappa B nuclear transcription factor (NFkB) and activator protein-1 (AP1), whereas inflammation causes oxidative stress, since ROS production is the result of activated immune cells (8,9,35). It is known that a single exercise session is able to induce ROS formation and, subsequently, lead to oxidative stress (9). However, when the physical exercise stressor stimulus is chronically maintained with training, the adaptations generated increase the resistance to ROS resulting from lipid peroxidation and increase the antioxidant defense (29). This is because endurance training leads to phenotypic and physiological responses that include activation of mitochondrial biogenesis, fiber type transformation and angiogenesis. Together, they increase the muscle’s capacity of aerobic metabolism and its resistance to fatigue (33,34). Thus, the adaptations related to redox balance (production of free radicals versus antioxidant defense) seem to be modulated by the production of free radicals, with consequent activation of the antioxidant defense system.

In the L30 situation, the increase in IL-6 level without an increase in sTNFR1 level after exercise could initially suggest an anti-inflammatory effect of this exercise. However, since this response was not accompanied by a significant alteration in MDA, CAT, and SOD levels, this exercise intensity probably was unable to generate the physiologic stimulus necessary for the adaptations. In healthy subjects, the acute increase in IL-6 levels after a single exercise session may be related to metabolic adjustments to the exercise intensity, and not necessarily to alterations in immune function (36).

The L45 exercise (isocaloric to M30) was studied to assess whether immune response and redox balance depend on the intensity–duration and/or energy expenditure of the activity performed. Hence, if we examine the responses of IL-6, sTNFR1, and MDA to the exercise session, it can be observed that their behavior was more similar, although smaller in magnitude, to the M30 situation than to the L30 situation. Despite the lack of a statistically significant alteration observed with L45 exercise when compared with M30, the IL-6 levels after exercise decreased by 13.5% in L45 and 11.7% in M30; the sTNFR1 levels increased by approximately 4% in L45 and 13.5% in M30; the MDA levels increased by 9% in L45 and 11% in M30. With L30, we observed an increase of 43.2% in IL-6 levels and a decrease of 3.4% in sTNFR1 levels and 3.3% in MDA levels, which are responses opposite to those found with L45 and M30 exercises.

The magnitude of the increase in plasma IL-6 levels depends on the duration and intensity of exercise, and on the muscle mass involved in the exercise (31,32,36). Evidence suggests that exercise duration is the determining factor responsible for more than 50% of the variation in the increase in IL-6 levels after exercise (37). However, this was not observed in the present study. As long-duration exercise frequently has a low intensity, the IL-6 response, as well as that of the other markers, may have been attenuated by the relatively low intensity (40% VO_2_peak) of L45 exercise. Very low-intensity exercises require a lower contractile response because they recruit less muscle mass than do the higher-intensity exercises. Thus, as the skeletal muscle is an important source of plasmatic IL-6, the recruitment of a smaller number of myofibrils may have been insufficient to increase IL-6 levels above baseline values despite the prolonged duration of 45 min.

This study has several limitations. First, the relatively small sample size consisting of few individuals with CHF of NYHA classes III and approximately 80% of the sample belonging to classes I and II present small limitations. Thus, it is not possible to apply these results to individuals with CHF of class III and IV, whose functional disability is higher. Second, this study lacks age- and body weight-matched controls. Third, we cannot say with certainty that intensity was the factor that most influenced the physiological adjustments to exercise because we did not evaluate the effects of moderate intensity exercise with longer duration (for 45 min, for example). Further, the study design does not allow us to infer on the long-term effects of the studied exercises. Future studies are necessary to confirm whether the loads imposed by different types of exercises would result in beneficial long-term adaptations to functionality, immune response, and oxidative stress. It will be also useful to analyze the cause-and-effect mechanisms involved in these adaptations.

In summary, we found that the acute responses to moderate-intensity physical exercise for 30 min (M30) initially provoked oxidative stress and inflammation. One hour after the exercise, there was an anti-inflammatory response and activation of the antioxidant defense system. Further, L45 exercise, although isocaloric to M30 and of the same intensity as L30, presented immunologic and redox balance adjustments intermediate to the other two situations. This could have occurred because the longer duration (45 min) of this activity was compensated for by its low intensity. Since L30 exercise imposed the smallest load on the immune system and redox balance, the adaptive responses that resulted were of lower intensity. These findings suggest that compared with the duration of exercise, the exercise intensity seems to have been an important factor of physiologic adjustments, as in acute oxidative stress and inflammation markers.
